# Supplementary material for: The Systems Biology Research Tool: evolvable open-source software
Source: BMC Syst Biol. 2008 Jun 29;2:55. doi: 10.1186/1752-0509-2-55 (PMC2446383; doi:10.1186/1752-0509-2-55)
Supplement: Additional file 1 — SBRT Archive. An archive of the current version of the Systems Biology Research Tool. [file 1752-0509-2-55-S1.zip › sbrt-1.4.0/doc/developers_guide/api/sbrt/shell/text/SimpleSetFormat.html]

SimpleSetFormat


|  |  |  |
| --- | --- | --- |
| **Overview** \| **Package** \| **Class** \| **Use** \| **Tree** \| **Deprecated** \| **Index** \| **Help** | |  |
| **PREV CLASS**   **NEXT CLASS** | **FRAMES**    **NO FRAMES**     **All Classes** |  |
| SUMMARY: NESTED \| FIELD \| CONSTR \| METHOD | DETAIL: FIELD \| CONSTR \| METHOD |  |


---


## sbrt.shell.text Class SimpleSetFormat<E>

```
java.lang.Object
  sbrt.shell.text.SimpleSetFormat<E>
```

**Type Parameters:**: `E` - the type of element.

**All Implemented Interfaces:**: Format, Formatter<java.util.Set<E>>, Parser<java.util.Set<E>>, SimpleFormat<java.util.Set<E>>

---

``` public class SimpleSetFormat<E> extends java.lang.Object implements SimpleFormat<java.util.Set<E>> ```

This interface is used to wrap `ListFormat` objects as `SimpleFormat`
objects.

**Author:**
:   This interface was written and documented by
    Jeremiah Wright while in the Wagner lab.

---

**Constructor Summary**

`SimpleSetFormat(LinkedHashSetFormat<E> setFormat)`
  
          Constructs a new simple set format from the provided set format.


| **Method Summary** | |
| --- | --- |
| `java.lang.String` | `format(java.util.Set<E> set)`             Returns a formatted string representation of the provided set. |
| `Formatter<E>` | `getElementFormatter()`             Returns the formatter for the elements of this set format. |
| `Parser<E>` | `getElementParser()`             Returns the parser for the elements of this set format. |
| `LinkedHashSetFormat<E>` | `getSetFormat()`             Returns the underlying set format. |
| `java.util.Set<E>` | `parse(java.lang.String set)`             Parses the provided string and returns its corresponding collection of elements as a set. |

**Methods inherited from class java.lang.Object**

`clone, equals, finalize, getClass, hashCode, notify, notifyAll, toString, wait, wait, wait`

**Methods inherited from interface sbrt.shell.text.Formatter**

`format`

**Constructor Detail**

### SimpleSetFormat

```
public SimpleSetFormat(LinkedHashSetFormat<E> setFormat)
```

:   Constructs a new simple set format from the provided set format.

    **Parameters:**: `setFormat` - the set format to be wrapped.


**Method Detail**

### format

```
public java.lang.String format(java.util.Set<E> set)
```

:   Returns a formatted string representation of the
    provided set.

    :   **Parameters:**: `set` - the set to be formatted. **Returns:**: a formatted string representation of the provided set.

---


### parse

```
public java.util.Set<E> parse(java.lang.String set)
```

:   Parses the provided string and returns its
    corresponding collection of elements as a set.

    :   **Specified by:**: `parse` in interface `Parser<java.util.Set<E>>`
    :   **Parameters:**: `set` - the string to be parsed. **Returns:**: the set of elements corresponding to the provided string.

---


### getSetFormat

```
public LinkedHashSetFormat<E> getSetFormat()
```

:   Returns the underlying set format.

    :   **Returns:**: the underlying set format.

---


### getElementFormatter

```
public Formatter<E> getElementFormatter()
```

:   Returns the formatter for
    the elements of this set format.

    :   **Returns:**: the formatter for the elements of this set format.

---


### getElementParser

```
public Parser<E> getElementParser()
```

:   Returns the parser for
    the elements of this set format.

    :   **Returns:**: the parser for the elements of this set format.


---


|  |  |  |
| --- | --- | --- |
| **Overview** \| **Package** \| **Class** \| **Use** \| **Tree** \| **Deprecated** \| **Index** \| **Help** | |  |
| **PREV CLASS**   **NEXT CLASS** | **FRAMES**    **NO FRAMES**     **All Classes** |  |
| SUMMARY: NESTED \| FIELD \| CONSTR \| METHOD | DETAIL: FIELD \| CONSTR \| METHOD |  |


---
